# Insights Into the Mineralogy and Surface Chemistry of Extracellular Biogenic S^0^ Globules Produced by *Chlorobaculum tepidum*

**DOI:** 10.3389/fmicb.2019.00271

**Published:** 2019-02-25

**Authors:** Cassandra L. Marnocha, Chandran R. Sabanayagam, Shannon Modla, Deborah H. Powell, Pauline A. Henri, Andrew S. Steele, Thomas E. Hanson, Samuel M. Webb, Clara S. Chan

**Affiliations:** ^1^Department of Biology, Niagara University, Lewiston, NY, United States; ^2^Department of Geological Sciences, University of Delaware, Newark, DE, United States; ^3^Delaware Biotechnology Institute, Newark, DE, United States; ^4^Geophysical Laboratory, Carnegie Institution for Science, Washington, DC, United States; ^5^School of Marine Science and Policy, University of Delaware, Newark, DE, United States; ^6^Department of Biological Sciences, University of Delaware, Newark, DE, United States; ^7^Stanford Synchrotron Radiation Lightsource, SLAC National Accelerator Laboratory, Menlo Park, CA, United States

**Keywords:** elemental sulfur, biomineralization, sulfur cycling, green sulfur bacteria, microbe–mineral interactions

## Abstract

Elemental sulfur (S^0^) is produced and degraded by phylogenetically diverse groups of microorganisms. For *Chlorobaculum tepidum*, an anoxygenic phototroph, sulfide is oxidized to produce extracellular S^0^ globules, which can be further oxidized to sulfate. While some sulfur-oxidizing bacteria (e.g., *Allochromatium vinosum*) are also capable of growth on commercial S^0^ as an electron donor, *C. tepidum* is not. Even colloidal sulfur sols, which appear indistinguishable from biogenic globules, do not support the growth of *C. tepidum*. Here, we investigate the properties that make biogenic S^0^ globules distinct from abiotic forms of S^0^. We found that S^0^ globules produced by *C. tepidum* and abiotic S^0^ sols are quite similar in terms of mineralogy and material properties, but the two are distinguished primarily by the properties of their surfaces. *C. tepidum*’s globules are enveloped by a layer of organics (protein and polysaccharides), which results in a surface that is fundamentally different from that of abiotic S^0^ sols. The organic coating on the globules appears to slow the aging and crystallization of amorphous sulfur, perhaps providing an extended window of time for microbes in the environment to access the more labile forms of sulfur as needed. Overall, our results suggest that the surface of biogenic S^0^ globules may be key to cell–sulfur interactions and the reactivity of biogenic S^0^ in the environment.

## Introduction

Globules of elemental sulfur (S^0^) are frequently produced as a required intermediate in the oxidation of sulfide to sulfate. The formation and degradation of S^0^ as an intermediate is special among lithoautotrophs and is facilitated by the many oxidation states of sulfur. While intracellular S^0^ deposition occurs in many sulfur-oxidizing taxa, the case of *extracellular* biogenic S^0^ deposition is intriguing in an environmental context, where it is common. In the environment, extracellular biogenic S^0^ is mobile, can serve as reactive surface, and could, in principle, be used by organisms other than the producing species. Yet in order to understand how these processes occur, it is critical to understand *what* biogenic S^0^ is. Chemical speciation of sulfur in globules produced by several taxa has been debated, complicated by the fact that many sulfur intermediates are short lived, highly reactive, and difficult to quantify in living systems. Thus, we investigated the properties of the extracellular S^0^ globules of a model sulfur-oxidizing bacterium *Chlorobaculum tepidum* in an effort to understand how these properties facilitate globule formation and degradation, and the persistence and reactivity of biogenic S^0^ in the environment.

Studies on several sulfur oxidizing bacteria (SOB), including *C. tepidum*, have come to a consensus on only a few basic properties of biogenic S^0^: that biogenic S^0^ is hydrophilic and that it must be labile (or at least, more labile than crystalline S^0^) to facilitate its enzymatic degradation (Steudel, 1989). The other properties of biogenic S^0^, and what distinguishes it from abiotic S^0^, are either unknown or contested (Pickering et al., 1998; Prange et al., 1999, 2002; George et al., 2008). This distinction between biogenic and abiotic S^0^ is important, as *C. tepidum* will not consume various forms of abiotic S^0^ (Hanson et al., 2016). These properties suggest that some yet unknown characteristic of biogenic S^0^ is required for its enzymatic degradation. This appears to be the case for S^0^ deposited intracellularly, where globules are enveloped by a proteinaceous globule envelope (Strohl et al., 1981; Pattaragulwanit et al., 1998; Prange et al., 2004). This envelope is composed of sulfur globule proteins (SGPs), and is thought to preserve sulfur in a reactive state and impart hydrophilic properties to the globule surface (Steudel et al., 1989). However, extracellular S^0^ producers like *C. tepidum* have no known homologs of SGP genes in their genomes (Shively et al., 2001). Thus it is unclear what differentiates biogenic S^0^ from abiotic S^0^ and makes it available for *C. tepidum*.

Here, we describe the results from work on extracellular biogenic S^0^ produced by *C. tepidum*. We found that globules are soft and flexible, yet solid biominerals composed of nanocrystalline α-S_8_ and enveloped by a recalcitrant organic coating. Globules produced by WT *C. tepidum* resist transformation to bulk crystals over time, compared with uncoated abiotic sols. Thus, these results have implications for the reactivity, aging, and persistence of biogenic S^0^ in the environment.

## Materials and Methods

### *Chlorobaculum tepidum* Cultures

*Chlorobaculum tepidum* strain WT2321 was used in all cultures and grown in Pf-7 medium (Chan et al., 2008) with a 177 kPa anaerobic headspace composed of 95% N_2_ and 5% CO_2_ passed through a heated copper scrubber. Cultures were inoculated to an initial density of 4 μg protein ml^-1^. In instances where cultures were grown with sulfide as the sole electron donor, thiosulfate and sulfide were omitted from Pf-7 to make sulfur-free Pf-7 (SF PF-7), and concentrated stocks of sulfide (Siefert and Pfennig, 1984) were used to amend SF Pf-7 to the approximately 2.5 mM concentrations. All culture media were buffered to pH 6.9–7.0 with 10 mM Bis-Tris-propane. Standard growth conditions were 47°C and 20 μmol photons m^-2^ s^-1^ from GE incandescent bulbs, as measured with a light meter equipped with a quantum PAR sensor (LI-COR). Primary cultures from cryo-stocks were grown under these conditions for 40–48 h, and then used to inoculate secondary cultures used in the subsequent analyses.

### Generation and Purification of S^0^

Cultures (0.5–0.6 L) of strain WT2321 were grown on sulfide-only Pf-7 (4–5 mM sulfide) in narrow-mouth screw-cap bottles with an open phenolic cap and butyl rubber septa at 30 μmol photon m^-2^ s^-1^ for 1–1.5 days until sulfide was no longer detectable by a qualitative assay: equal volumes of culture supernatant were mixed with 10 mM CuCl_2_, where the formation of a distinct gray precipitate indicated the presence of sulfide greater than 0.2 mM. Cultures were transferred into sterile 250 ml centrifuge bottles with o-ring sealing caps (Nalgene, Thermo Fisher Scientific). The S^0^ was pelleted through the sucrose by centrifugation at 6,000 × *g* (JS-13.1 rotor) for 50 min at 4°C. The supernatant was removed, and the resuspended pellet was centrifuged through 2.5 M sucrose two more times. Collected S^0^ was washed to remove sucrose by suspending in S-free Pf-7 and centrifuging at 17,500 × *g* (JS-13.1 rotor) for 5 min at 10°C; this step was repeated twice. S^0^ was suspended in SF Pf-7 and immediately distributed into aliquots for characterization studies; aliquots were stored at -80°C until use.

### X-Ray Diffraction

Bottle cultures of *C. tepidum* amended with 5 mM sulfide were incubated for 17 h, then concentrated using a series of low-speed (2,500 rpm) centrifugation and wash steps with S-free PF7. The resulting pellet contained cells, but was largely biogenic sulfur. The supernatant was decanted and the sulfur-cell pellet was dried down on a low background slide under vacuum in the loading dock of an anaerobic chamber. The slide was then analyzed with a Bruker D8 XRD using a monochromatic Cu Kα1 source. Scans were taken from 10 to 50 2𝜃 at 1 s intervals and in increments of 0.05°. Scans were typically allowed to run continuously overnight.

### Spectroscopy

Raman spectroscopy was conducted at the Carnegie Institution of Science (Washington, DC, United States) using a Witec α-scanning near-field optical microscope that incorporates a confocal Raman spectroscopy imaging. The excitation source was a frequency-doubled solid-state YAG green laser (532 nm) operating between 0.3 and 1 mW output power. Objective lenses used included a 100× and 60× long working distance. Raman signal were collected using a 600 lines/mm grating on a Peltier-cooled Andor EMCCD chip with a EMCCD Gain of 100. Spectra were collected over the range 0 to 3600 cm^-1^ and averaged using 1 s integration time. The lateral resolution of the instrument is about 380 nm, with a focal plane depth of ∼700 nm. For α-S_8_ standard we used the commercial precipitated sulfur (purity >99%, Thermo Fisher Scientific). Drops of living cultures were deposited on a microscope slide and covered by a microscope cover slip. The edges of the cover slip were immediately sealed to the slide using nail polish and the samples were analyzed within minutes after the slide preparation. Crystalline standards were measured directly on glass coverslips.

Cultures for NanoIR were prepared with a section of a sterile glass slide inside the tube, onto which cells and sulfur (and ultimately, remnant particles) adhered. The glass slide was removed from the tube, allowed to dry, and was examined in a nanoIR2 system in resonance-enhanced mode equipped with a QCL laser as the IR source. The spectra were acquired over the 1000–1800 cm^-1^ range with a spectral resolution of 2 cm^-1^.

Infrared spectra were obtained using a Spectrum 2 FTIR spectrometer (PerkinElmer, Waltham, MA, United States) using an Attenuated Total Reflectance (ATR) attachment. Spectra were collected from 400 to 4000 cm^-1^, at a 1 cm^-1^ scan resolution. Contributions from atmospheric CO_2_ and H_2_O were subtracted from spectra using a correction within the Spectrum software. Purified biogenic S^0^ suspended in S-free Pf7 medium was centrifuged into a pellet, then dried down onto a slide and collected as a powder for analysis on the instrument.

### Atomic Force Microscopy

A Bruker Catalyst AFM was used to measure the Young’s modulus of S^0^ globules. Bruker MCLT “F” AFM probes with nominal spring force constant *k* = 0.5 N/m were used in non-resonant tapping mode called, PeakForce Quantitative Nanomechanical imaging, that provides height, adhesion, deformation, and modulus “maps” of the scan area. Prior to imaging, the spring force constant for each probe was determined using thermal tuning and varied between 0.9 and 1.3 N/m. In order to estimate the S^0^ moduli, PDMS (polydimethylsiloxane) with a known elastic modulus of 3.5 MPa was used to calibrate the instrument as follows, following the guidelines of Bruker. First, the PeakForce setpoint and PeakForce amplitude was set to 1.0 nN and 300 nm resulting in an average PDMS deformation of 5 nm. Then, the AFM tip radius value was adjusted so that the average measured modulus was 3.5 MPa. After the calibration procedure, the Young’s modulus of other samples can be determined by changing the PeakForce setpoint and/or PeakForce amplitude to produce an average 5 nm sample deformation, while keeping the tip radius unchanged from the PDMS calibration. Typical values of the setpoint and amplitude ranged from 1.0 to 4.0 nN and 100 to 300 nm, respectively. AFM scans were 5.0 μm × 5.0 μm (256 pixels × 256 pixels) acquired with a scan rate of 1 kHz. Finally, to estimate the Young’s moduli of individual S^0^ globules adsorbed onto ACLAR substrates, a region of interest was selected within a globule and the pixel-by-pixel modulus values were depicted as box plots.

Abiotic S^0^ sols were prepared for AFM by incubating a strip of ACLAR in a tube of uninoculated media amended with sulfide. Prior to AFM imaging, the ACLAR was removed from the tube and exposed to air for approximately 2 h to generate sols on the surface of the ACLAR substrate. Residual media covered the strip and was replenished periodically to avoid complete evaporation. Sols analyzed by AFM averaged 692 nm in diameter, comparable to the size of 5-h biogenic globules (862 nm), but smaller than 8-h biogenic globules (1.18 μm).

### Cryo-Scanning Electron Microscopy and Transmission Electron Microscopy

Samples were prepared for cryo-scanning electron microscopy (cryoSEM) either on filters or on ACLAR (Honeywell/Allied Signal), a fluoropolymer film. Filtered samples were prepared by carefully pipetting culture onto 0.2 μm pore size polycarbonate filters (Millipore) and gently washed with deionized water. For ACLAR samples, strips of ACLAR were autoclaved and added to tubes, which were then filled with S-free Pf7 medium in an anaerobic chamber. Tubes were then stoppered and sealed, followed by headspace exchange and pressurization to 10 p.s.i. (∼69 kPa) with 5% CO_2_ + 95% N_2_ gas passed through heated copper. At least 24 h prior to inoculation, ACLAR tubes were amended with 2.5 mM sulfide. ACLAR tubes were then inoculated, with care taken to inject inoculum over the ACLAR strip. Tubes were incubated as described above, but secured horizontally in the water bath. Following incubation, the ACLAR strip was removed and cut to size for imaging. Samples were mounted onto specimen holders with Tissue Freezing Medium (Electron Microscopy Sciences). Samples were plunged into liquid nitrogen slush and transferred under vacuum to the Gatan Alto 2500 cryo chamber at a temperature of -120°C. Samples were then sublimated for 10 min at -90°C followed by cooling to -120°C. A thin layer of gold-palladium was sputtered onto the samples. The samples were then transferred into a Hitachi S-4700 field-emission scanning electron microscope for imaging.

TEM was performed on whole mounted samples on lacey carbon-coated grids using a Zeiss Libra 120 transmission electron microscope equipped with an in-column Omega energy filter. Images were acquired at 120 kV using a Gatan Ultrascan 1000 CCD. EELS spectra were acquired for nitrogen and carbon at the respective K edges (397 and 283 eV), and analyzed using Gatan Digital Micrograph software.

### Light and Fluorescence Microscopy

Phase contrast, differential interference contrast (DIC), and fluorescence microscopy were performed on a AxioImager Z1 microscope (Zeiss) with a 40× EC Plan NeoFluar lens and/or 100× Plan Apochromat oil immersion lens (Zeiss). Live cultures of *C. tepidum* and abiotic sols produced by exposing Pf-7 medium to air were stained with the lipophilic styryl dye FM 1-43FX (Molecular Probes) by mixing an aliquot of culture, Vectashield (Vector Laboratories), and the FM dye on a slide in a ratio of 8:1:1 by volume.

### X-Ray Microspectroscopy

X-ray fluorescence images and spectroscopy were collected at the Stanford Synchrotron Radiation Lightsource (SSRL) using beam line 14–3. The incident x-ray energy was obtained using a Si (111) double crystal monochromator with the Stanford Positron Electron Accelerating Ring (SPEAR) storage ring containing 500 mA at 3.0 GeV in top-off mode. The energy of the monochromator was calibrated to the pre-edge peak of sodium thiosulfate and set to be 2472.02 eV. The Kα fluorescence line of the sulfur was monitored with a silicon drift Vortex detector (Hitachi) using Xspress3 pulse processing electronics (Quantum Detectors). The microfocused beam of 5 μm × 5 μm was provided by a Cr-coated Kirkpatrick-Baez mirror pair (Xradia Inc.) Standards and samples of biogenic S^0^ globules were placed on a nucleopore membrane filter and placed at 45° to the incident x-ray beam. Data were normalized and processed using standard methods in the SIXPACK analysis package (Webb, 2005). Fitting of the sample data to standards was performed using linear combination fitting.

## Results and Discussion

### Alpha-Cycloocta Sulfur (α-S_8_) in Biogenic Globules and Abiotic Sols

**FIGURE 1:**
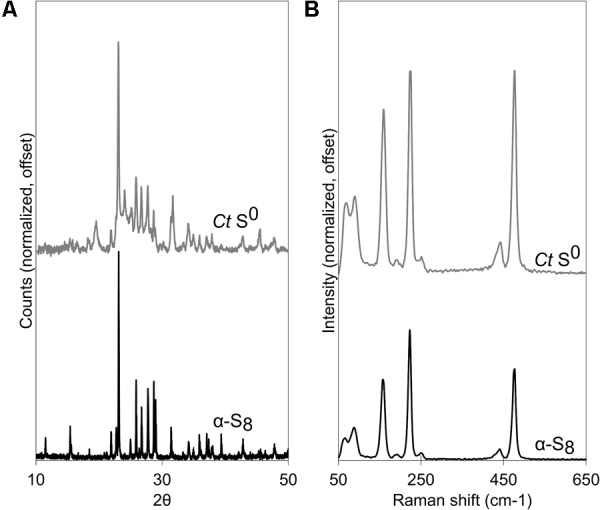
*Chlorobaculum tepidum* biogenic S^0^ globules (*Ct* S^0^) compared to a standard of bulk crystalline α-S_8_. **(A)** XRD patterns, with peak broadening in S^0^ globules. **(B)** Raman spectra.

Biogenic S^0^ globules produced by *C. tepidum* are at least in part composed of α-S_8_. XRD patterns of biogenic globules collected at the transition between S^0^ production and S^0^ consumption (i.e., when HS^-^ in the medium had been depleted, or about 17 h post-incubation in a 5 mM HS^-^ culture) matched well with database diffraction patterns for α-S_8_ (Figure 1A). Peak broadening in XRD diffractograms suggests that the biogenic S^0^ is nano- or poorly crystalline (Figure 1A). Using the Debye-Scherrer equation, domain sizes of α-S_8_ crystals in globules were estimated to be between 42 and 48 nm. Sulfur K-edge XAS spectral fits of globules included both Weimarn sols (21%) and solid α-S_8_ (47%), further supporting the inference that globules are at least partially crystalline (Supplementary Figure S1). Likewise, Raman spectroscopy of globules during S^0^ production also showed the presence of α-S_8_ when compared against a commercial S^0^ standard (Figure 1B). Overall, this suggests that S^0^ globules produced by *C. tepidum* are composed of aggregates of nanocrystalline α-S_8_.

Considerable work has been done to determine the chemical structure of sulfur in biogenic globules (Prange et al., 1999; Pasteris et al., 2001; Pickering et al., 2001; George et al., 2008; Berg et al., 2014). For *C. tepidum*, globules appear to be mainly α-S_8_, which follows the findings from Pasteris et al. (2001) and George et al. (2008) for *C. tepidum* and other sulfur globule producers like *Beggiatoa alba* and *Thiomargarita namibiensis* (Pasteris et al., 2001). Some of the controversy surrounding speciation of sulfur in globules harkens back to pervasive descriptions of “liquid” or “liquid-like sulfur” (Hageage et al., 1970). Indeed, if sulfur in globules were composed of organic residues and polysulfides, this might account for liquid-like amorphous sulfur within globules. The generation of a diffraction pattern, however, suggests a considerable proportion of the globule is crystalline. Pasteris et al. (2001) also showed “microcrystalline solid elemental sulfur” in Raman spectra of globules. In both Raman spectroscopy and XRD, peak broadening occurred, suggesting that the α-S_8_ in globules occurs in nanocrystalline form. This is in agreement with the domain sizes calculated for biogenic S^0^ globules produced by *C. tepidum*.

Abiotic sols, such as Weimarn or Raffo sols, will eventually transform into bulk crystalline α-S_8_ (Steudel, 2003; Garcia and Druschel, 2014). Following their formation, a transformation will begin from amorphous sulfur, to loosely ordered, to nanocrystalline, and eventually to bulk crystalline α-S_8_, in some cases on the timescale of hours (Kamyshny and Ferdelman, 2010; Garcia and Druschel, 2014). Thus, for abiotic sols, the degree of sulfur crystallinity appears to exist on a continuum, with freshly precipitated sulfur less crystalline than sulfur that has aged. The observed mixture of nanocrystalline and amorphous sulfur in biogenic globules suggests biogenic S^0^ may age similarly, but the conditions in which aging occurs, and whether it is hastened or slowed, are unclear.

The significance of amorphous vs. nanocrystalline vs. crystalline S^0^ lies in the bioavailability of those forms. Other sulfur oxidizers appear to preferentially consume sulfur with structures that are more bioavailable. In the chemoautotrophic sulfur oxidizer *Acidithiobacillus albertensis*, for example, the crystal microstructure of S^0^ affects oxidation rates (Laishley et al., 1986). A mix of polymeric and orthorhombic sulfur had the lowest oxidation rate in *A. albertensis* cultures, which was attributed to alterations of the orthorhombic S_8_ crystal lattice by polymeric sulfur (Laishley et al., 1986). Likewise, for purple sulfur bacterium *Allochromatium vinosum*, the polymeric component of commercial sulfur was more bioavailable than the cyclo-octasulfur component (Franz et al., 2007). Examples like these highlight the influence of both sulfur speciation and crystal structure in the bioavailability of elemental sulfur.

Despite the differences observed in the bioavailability of various forms of sulfur described above, globules produced by *C. tepidum* appear to be similar to sulfur produced by other SOB, as well as to abiotic sols with respect to sulfur speciation and degree of crystallinity. Nevertheless, preferential growth on different forms of sulfur (e.g., *C. tepidum*’s inability to grow on commercial S^0^) suggests that some differences between abiotic S^0^ and biogenic S^0^ are unaccounted for. Though mechanisms of formation may be similar between abiotic and biogenic S^0^, the question of what confers and maintains lability to globules remains.

### Material Properties of Biogenic Globules and Abiotic Sols

**FIGURE 2:**
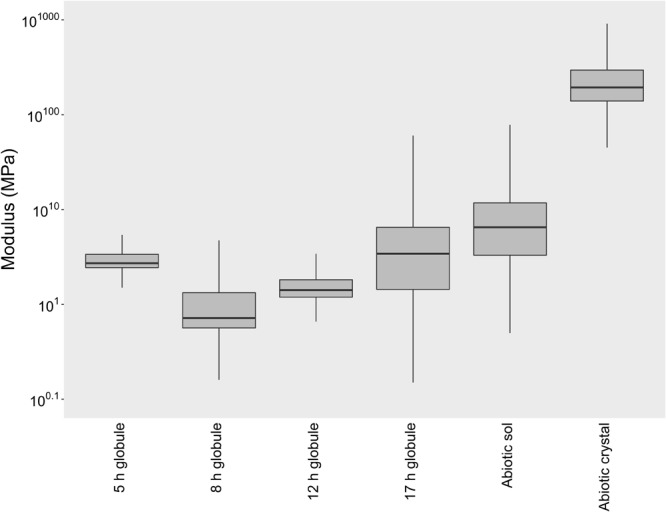
Elastic modulus box plots of biogenic S^0^ globules in both production (5, 8 h post-inoculation) and degradation stages (12, 17 h post-inoculation), abiotic S^0^ sols, and abiotic S^0^ crystals. The horizontal line within each box indicates the median, while boundaries of the boxes indicate the 25th and 75th percentiles. Whiskers indicate the highest and lowest values of the data.

To determine if differences exist in the physical properties of biogenic and abiotic S^0^, we used AFM to measure the elastic modulus of globules and sols. Elastic modulus is a measure of the stiffness of a material (stress-to-strain ratio). Surprisingly, we found that globules are soft and flexible solids, much more so than their bulk crystalline counterparts (Figure 2). The elastic moduli of globules ranged between 0.5 and 5 MPa (Figure 2), equivalent to the stiffness of materials such as PDMS, polyacrylamide gels, or small strain rubber. Globules that were partially degraded were similarly soft, but with a much wider range of observed moduli. In a few cases, partially degraded globules reached upward of 100 MPa. This range could be a reflection of a more heterogeneous globule interior as sulfur becomes more crystalline with time, either as a result of preferential consumption of amorphous phases or ongoing ripening of S^0^. Nevertheless, biogenic S^0^ globules at any stage of production or degradation were consistently softer (less stiff) than bulk abiotic S^0^ crystals, which ranged from 100 to 1000 MPa and typically exceeded 250 MPa.

Abiotic S^0^ sols, produced by the exposure of Pf-7 medium to air, were similarly soft and solid sulfur. On average, abiotic sols tended to have a higher modulus (median modulus of 5.5 MPa) than biogenic S^0^ globules (median moduli between 0.7 and 3.3 MPa), but were still considerably softer than abiotic S^0^ crystals (median modulus of 202.5 MPa; Figure 2). Since abiotic S^0^ crystals were prepared by the same process of forming the sols (exposure of sulfide-amended medium to air), but over a longer period of time, the crystals can be thought of as the natural end state of α-S_8_ sols, which apparently become more and more ordered over time until they can be classified as bulk crystalline.

**FIGURE 3:**
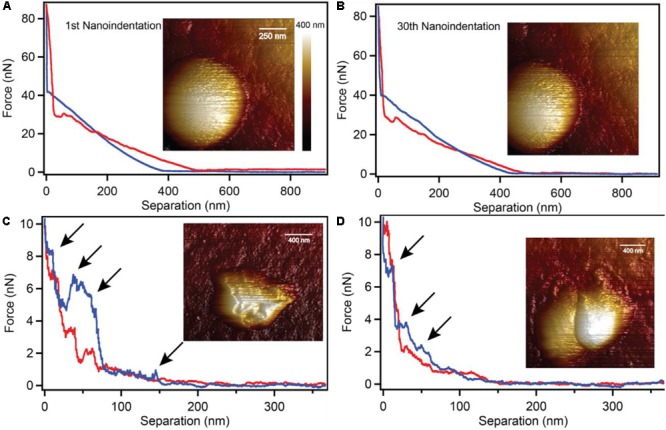
Nano-indentation of intact and partially degraded biogenic S^0^ globules. Intact S^0^ globules **(A)** withstood 30 repeated nano-indentations at 80 nN of force without any detectable defects or damage **(B)**. Partially degraded globules were easily damaged at forces of only 1–2 nN (**C,D**; arrows indicate damage).

Biogenic S^0^ globules are also remarkably flexible during the globule production stage. We used a nano-indentation AFM technique to test the structural soundness of S^0^ globules. Surprisingly, we found that the AFM tip was able to completely penetrate the globule through its center with a force ramp from 0 to 40 nN before hitting the underlying substrate (Figure 3A). These forces are strong enough to break viral capsids (Michel et al., 2006; Cuellar et al., 2010), indent and potentially puncture bacterial cells (Suo et al., 2009; Vadillo-Rodriguez et al., 2009; Baniasadi et al., 2014), damage bacterial nanowires (Leung et al., 2011), and flatten outer membrane vesicles (Ovidiu et al., 2003). Repeated indentations showed that the globule was not damaged physically or internally; the same amount of force was required to indent the globule consistently through all 30 indentations in the same location (Figure 3B). This suggests that despite being solid, the globule is composed of weak or pliable bonds that allow for repeated elastic deformation. Globules that had been partially consumed by *C. tepidum* (i.e., collected from cultures where sulfide had been depleted and where cells then began to oxidize sulfur) did not exhibit the same flexibility. Instead, the partially degraded globules were irreparably damaged at 1–10 nN, evident by the stepped decreases in the force-separation plots and the non-overlapping extension and retraction curves (Figure 3C,D).

The observations of soft S^0^ globules and sols suggests two things: (1) the precipitation of α-S_8_ by either abiotic or biogenic means occurs via roughly equivalent mechanisms; and (2) over time, sulfur (S_8_) becomes gradually more ordered, resulting in a greater proportion of stiffer, crystalline sulfur.

### Surface Characterization of Sulfur

**FIGURE 4:**
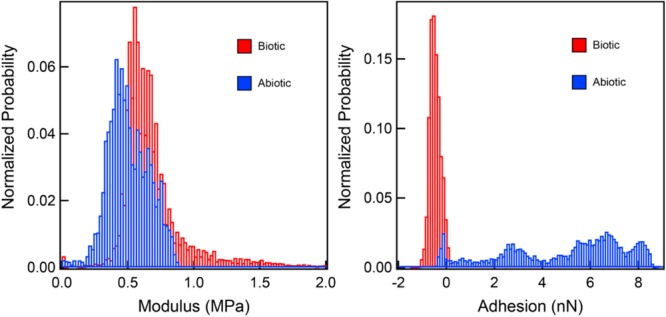
Elastic modulus and adhesion profiles of interactions between a silicon nitride AFM tip and both biogenic S^0^ globules and abiotic S^0^ sols. While elastic moduli are comparable between the two, the biogenic S^0^ globules are slightly repulsed by the AFM tip, while abiotic sols show a range of attractive forces.

Our characterization of biogenic S^0^ globules and abiotic S^0^ sols show that they are mineralogically and physically quite similar. However, biogenic S^0^ does not convert rapidly to S^0^ crystals like abiotic S^0^ sols do, even when exposed to air. In fact, biogenic S^0^ globules produced by *C. tepidum* resist complete conversion to micron-sized crystals when exposed to air for upward of 3 weeks, whereas abiotic sols can convert in as little as 48 h. Thus, we sought an explanation for the differences in ripening observed between the two, given that their physical properties and chemical structures appear virtually identical. During the AFM examination of the material properties of globules and sols, we found that while both are indistinguishable by elastic modulus and morphology, they have distinct adhesion profiles when interacting with the AFM tip (Figure 4). Thus, one of the central distinctions between *C. tepidum*’s biogenic S^0^ globules and abiotic S^0^ sols appears to be restricted to the surface of the particles.

**FIGURE 5:**
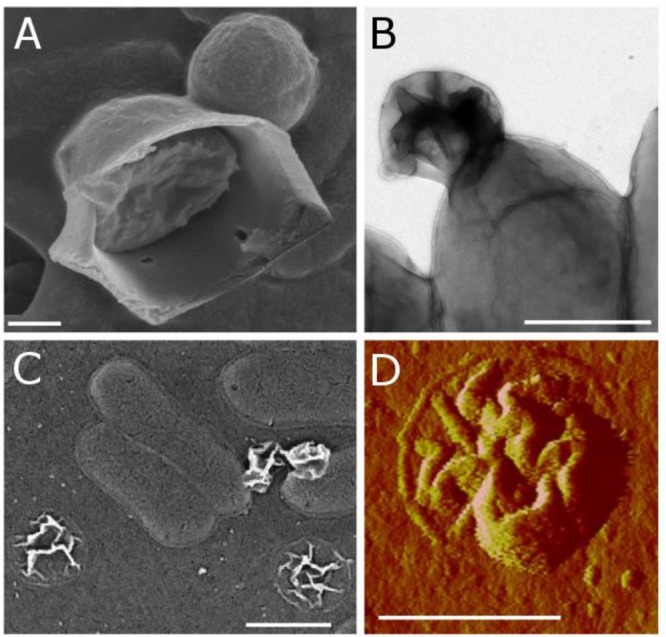
Biogenic S^0^ globule coatings observed in **(A)** SEM as a ruptured globule with interior sulfur present; **(B)** TEM as a remnant particle attached to a cell; **(C)** SEM as remnants on ACLAR substrate both attached to and apart from cells; and **(D)** AFM (peak force error) as a remnant particle.

Using a combination of microscopy techniques, we found that *C. tepidum*’s S^0^ globules appear to be enveloped in a thin, flexible coating. This coating was first observed as a remnant particle in cultures of *C. tepidum* that had completed a production/degradation cycle of S^0^ globules. Flattened, wrinkled particles (termed “remnants” hereafter) were consistently observed in *C. tepidum* cultures in electron microscopy and AFM (Figure 5A–C). Globules generally progress from full and roughly spherical, to semi-collapsed and irregular, and finally to the remnant stage. These remnants are approximately 20 nm thick, likely representing a double layer of the collapsed coating and appear in roughly the same frequency as globules at earlier time points, thus suggesting that every globule likely leaves a remnant. In time-lapse microscopy, the transition from globules to remnant can be directly observed. Remnants occur in late culture stages, when all sulfur should be oxidized to sulfate, and globules should be entirely consumed.

**FIGURE 6:**
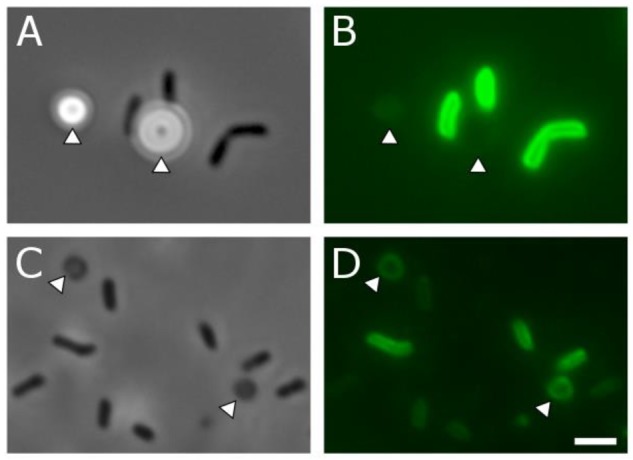
Phase contrast **(A,C)** and corresponding fluorescence microscopy **(B,D)** of *C. tepidum* and S^0^ globule coatings and remnants. **(A,B)** Cells (dark rods) and S^0^ globules (arrowheads) in the globule production stage, 4 h after inoculation. Faint fluorescent outlines from the FM membrane dye can be seen in **(B)**. **(C,D)** Cells (dark rods) and globule remnants (arrowheads) from a 48-h culture in which all sulfur has been depleted. Fluorescence from the FM membrane dye can be observed throughout the disk of the remnant particles in **(D)**. Scale bar is equal to 2 μm. Quantitative data for globule fluorescence is available in Supplementary Figure S3.

To further examine the globule coating, we employed a range of microscopy techniques. Modified cryo-scanning electron microscopy of biogenic S^0^ globules resulted in their rupture, showing evidence of distinct surface and core components (Figure 5D). A membrane dye, FM 1-43FX, was used to determine if the surface layer was present at all stages of globule growth and consumption. FM dyes are amphipathic molecules with positively charged hydrophilic head groups and hydrophobic tails. They are typically used in membrane studies, where they will fluoresce upon insertion into the outer leaflet of membranes and are expected to selectively associate with negatively charged phospholipids (Barák and Muchová, 2013), though they may also bind electrostatically. In biogenic S^0^ globules, this fluorescence was localized to the outer “rim” of globules, again suggesting a coating, though it is possible that the dye could not penetrate beyond the outermost portions of the coating. In contrast, remnants fluoresced throughout their disk, likely because they lie flat on the substrate and are, in whole, composed of material that the FM dye is likely to interact with. Larger, and thus, “older” globules displayed greater fluorescence, suggesting that the coating accumulates with time (Figure 6). However, larger globules observed at the same time point showed greater fluorescence than their smaller counterparts. Taken together, this may suggest that passive adsorption of organics is responsible for the coating, with a longer exposed duration to extracellular organics resulting in a greater concentration of organics at the globules surface, along with larger globules in general having greater surface area onto which organics might adsorb. Abiotic sulfur sols, including those incubated in spent medium for 5 h, did not fluoresce when stained with FM 1-43FX. An attempt to form abiotic sols in spent medium was made, but no visible sols were formed. This may be a result of slowed formation or reduced particle size because of surfactants in the spent medium (Garcia and Druschel, 2014). It should be noted that, on occasion, crystalline S^0^ within WT cultures was stained by the FM dye. It is possible that this fluorescence was caused by contaminating cell material or by the transformation of globules to crystals (and inclusion of the coating material) prior to globule degradation. Indeed, globules have been observed at intermediate stages of transformation from spherical globule to the classic bipyramidal crystal of α-S_8_ (Supplementary Figure S2), and self-assembly of carbon/sulfur microstructures (including envelopes) has been recently demonstrated for abiotic S^0^ (Cosmidis and Templeton, 2016).

### The Biogenic S^0^ Coating Is Composed of Proteins and Polysaccharides

**FIGURE 7:**
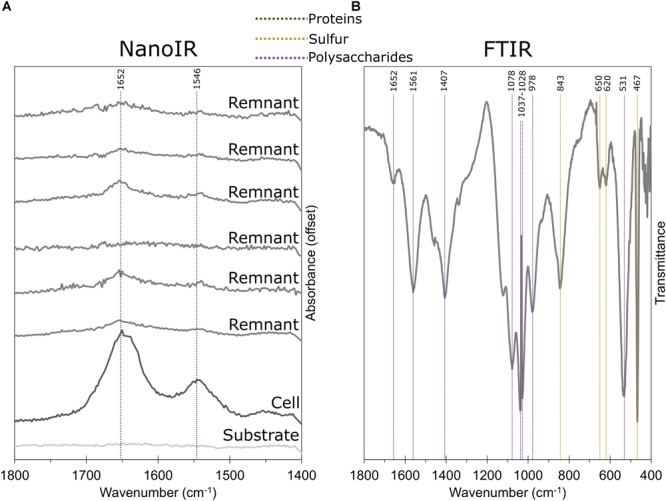
IR spectra of remnants and globules. **(A)** NanoIR spectra of a *C. tepidum* cell, S^0^ globule remnants, and the substrate. Amide bands indicative of protein are highlighted. **(B)** FTIR spectra of intact, purified biogenic S^0^ globules with amides, polysaccharide (e.g., C-O-C rings), and sulfur compounds (S-S stretch, S-OR esters) highlighted. Notably, FTIR data lacked characteristic bands for lipids, including 1739 cm^-1^ for membrane lipids (shown) and 3015 cm^-1^ for unsaturated fatty acids (not shown).

To determine the composition of the coating, we used a range of spectroscopy techniques on both intact globules and remnant particles. Carbon and nitrogen were observed in remnants using TEM/EELS, and N:C ratios showed that remnants were enriched in carbon relative to *C. tepidum* cells (Table 1). While the presence of nitrogen suggested protein as a component of the remnant, the enrichment of carbon also suggested a non-protein component. This is consistent with ToF-SIMS analyses of purified S^0^ globules that detected amino acid fragments and globule-associated proteins (Hanson et al., 2016). NanoIR was also used to further investigate the organic composition of remnants. Strong amide bands were observed in the remnants (Figure 7A), supporting the conclusions of the TEM/EELS analysis. Due to the nature of sample preparation and the limitations of the instrument, however, we were unable to identify bands that would confirm or repudiate the presence of other organics, such as lipids or polysaccharides, although polysaccharides were suspected and could account for the enrichment in carbon observed in the EELS data. FTIR was then used on purified samples of biogenic S^0^ from the C3 mutant strain of *C. tepidum* (Hanson et al., 2016). The globules showed bands indicative of protein, polysaccharide, and sulfur, but were notably lacking key bands suggestive of lipids (Figure 7B).

**Table 1:** Nitrogen/carbon ratios of cells, remnant particles, and a BSA protein standard from TEM/EELS data.

Sample	N/C
Lacy carbon	0
Remnant 01	0.04
Remnant 02	0.04
Remnant 03	0.04
Remnant 04	0.09
Remnant 05	0.1
Remnant 06	0.11
Remnant 07	0.12
Cell 01	0.13
Cell 02	0.13
BSA	0.18

There are a few possibilities for the role of this recalcitrant organic coating. The solubility of sulfur in water is low, with measured values between 19 and 30 nM S_8_ (Boulegue, 1978; Kamyshny, 2009; Wang and Tessier, 2009). This low solubility makes it difficult to access sulfur as an extracellular electron donor. However, neutral surfactants have been shown to increase solubility dramatically (Steudel, 2003). In intracellular S^0^ globules, the function is achieved by the SGP envelope (Steudel, 1996). For those S^0^ globule producers that do not produce SGP envelopes, a similar process may occur via passive adsorption of organics. Sulfur sols and globules should be capable of attracting biomolecules such as lipids and proteins, and indeed, adsorption of polar organic compounds onto biologically produced elemental sulfur has been observed (Jones and Benson, 1965; Cosmidis and Templeton, 2016). The result of such adsorption could hinder crystallization and confer a hydrophilic surface to otherwise hydrophobic elemental sulfur (Steudel and Albertsen, 1999). Based on contact angle measurements (data not shown), *C. tepidum* S^0^ globules are hydrophilic, especially when compared against abiotic sulfur. Likewise, while coarsening rates of abiotic sols increase with temperature, surfactants are able to slow coarsening rates and are particularly effective at the temperatures in which *C. tepidum* experiences optimal growth (Garcia and Druschel, 2014). Weimarn sols have also been shown to be stabilized in the presence of surfactants and biopolymers, slowing their crystallization and sedimentation for nearly twice as long as unstabilized sols (Steudel, 2003). For *C. tepidum*’s biogenic S^0^ globules, the organic coating may function similarly.

Our previous work showed that degradation of biogenic S^0^ globules could occur at-a-distance from *C. tepidum* cells (Marnocha et al., 2016). This process is likely facilitated by soluble intermediates like polysulfides breaking down the sulfur chemically. Because degradation at-a-distance appears to be just as important as degradation by attached cells, the reactivity of globules over time is an important consideration. The presence of the organic coating may play a role in maintaining the reactivity of biogenic sulfur, and perhaps be a key determinant of the persistence of extracellular biogenic sulfur in the environment.

The stabilization of biogenic S^0^ as poorly crystalline or nano-crystalline sulfur would make it more bioavailable for cells. This is in support of the observations of *C. tepidum*’s inability to grow on abiotic sulfur substrates (Hanson et al., 2016), where one would expect a more rapid aging process for abiotic sulfur leading to a less bioavailable bulk-crystalline sulfur. The coating could also account for our observations of globular sulfur persisting in preparations of purified biogenic S^0^, even in the presence of O_2_.

In the environment, this function may be more or less important depending on availability of sulfide. If a sulfide flux is intermittent, it may be more important to slow the crystallization of biogenic S^0^ globules so that they might be used as an alternative electron donor in the absence of sulfide.

## Conclusion

In sulfidic environments, especially those with localized or periodic exposure to O_2_, biogenic and abiotic sulfur may be produced simultaneously, although biogenic sulfide oxidation should outcompete abiotic sulfide oxidation (Luther et al., 2011). The byproduct of either sulfide oxidation process is elemental sulfur, occurring as particles of comparable size, morphology, and sulfur speciation. Elemental sulfur produced by *C. tepidum* is distinguished from other abiotic elemental sulfur sols in that it is surrounded by an organic coating of protein and polysaccharide, which may impart hydrophilic properties onto the surface of the globule, make S^0^ more reactive and bioavailable, and slow the transformation into a bulk crystalline state. As numerous studies have shown, the more crystalline the sulfur, the less bioavailable (Laishley et al., 1986; Franz et al., 2007; Hanson et al., 2016). In the environment, this would mean that biogenic sulfur would remain more accessible to sulfur oxidizers (and perhaps even sulfur reducers), and thus an alternative electron donor (or acceptor) would still be available even if sulfide (or sulfate) was absent.

Whether this is adaptation or incidental is unclear. Additionally, it is not known at this time if other extracellular sulfur producers have a similar organic coating. If having an organic, non-SGP coating is a widespread characteristic of extracellular sulfur globules, the association between organics and elemental sulfur could have far-reaching implications in environmental microbiology, biogeochemical cycling, and the preservation of organic material in the rock record.

## Author Contributions

CC and TH conceived of and directed the study. CS carried out portions of AFM, and processed and interpreted the AFM data. SM carried out TEM and EELS. DP performed the SEM imaging. PH and AS performed the Raman spectroscopy. SW performed the x-ray microspectroscopy. CC helped to draft the manuscript. CM carried out all other analyses and wrote the manuscript. All co-authors contributed to the editing of the manuscript.

## Conflict of Interest Statement

The authors declare that the research was conducted in the absence of any commercial or financial relationships that could be construed as a potential conflict of interest.
