# Erratum: Insights Into the Mineralogy and Surface Chemistry of Extracellular Biogenic S^0^ Globules Produced by *Chlorobaculum tepidum*

**DOI:** 10.3389/fmicb.2019.02237

**Published:** 2019-09-24

**Authors:** Frontiers Production Office

**Affiliations:** Frontiers Media SA, Lausanne, Switzerland

**Keywords:** elemental sulfur, biomineralization, sulfur cycling, green sulfur bacteria, microbe–mineral interactions

Due to a typesetting error, the citations for “Steudel, 2003” were replaced with question marks. Corrections have been made in the **Results and Discussion** section, sub-section **Alpha-Cycloocta Sulfur (a-S8) in Biogenic Globules and Abiotic Sols**, paragraph three, and sub-section **The Biogenic S0 Coating Is Composed of Proteins and Polysaccharides**, paragraph two.

The publisher apologizes for this error. The original article has been updated.

