# Supplementary material for: Insights Into the Mineralogy and Surface Chemistry of Extracellular Biogenic S0 Globules Produced by Chlorobaculum tepidum
Source: Front Microbiol. 2019 Feb 25;10:271. doi: 10.3389/fmicb.2019.00271 (PMC6398422; doi:10.3389/fmicb.2019.00271)
Supplement: Supplementary file 1 [file Data_Sheet_1.docx]

**Supplemental Information**

**
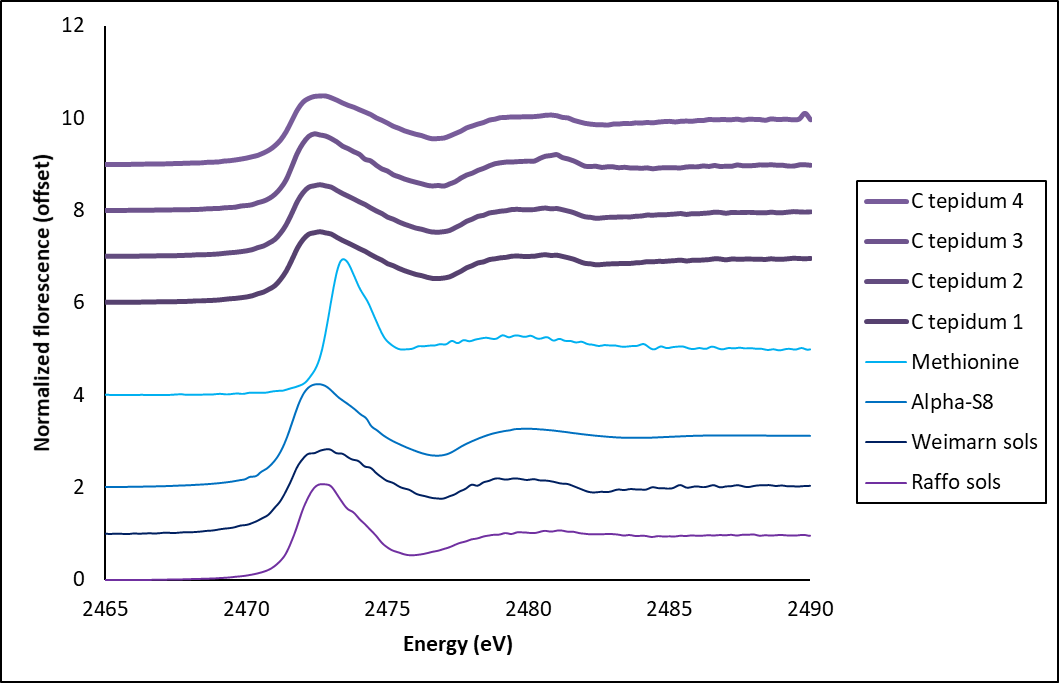
**

Figure S1. XAS spectra of *Cba. tepidum* S^0^ globules compared against sulfur standards.


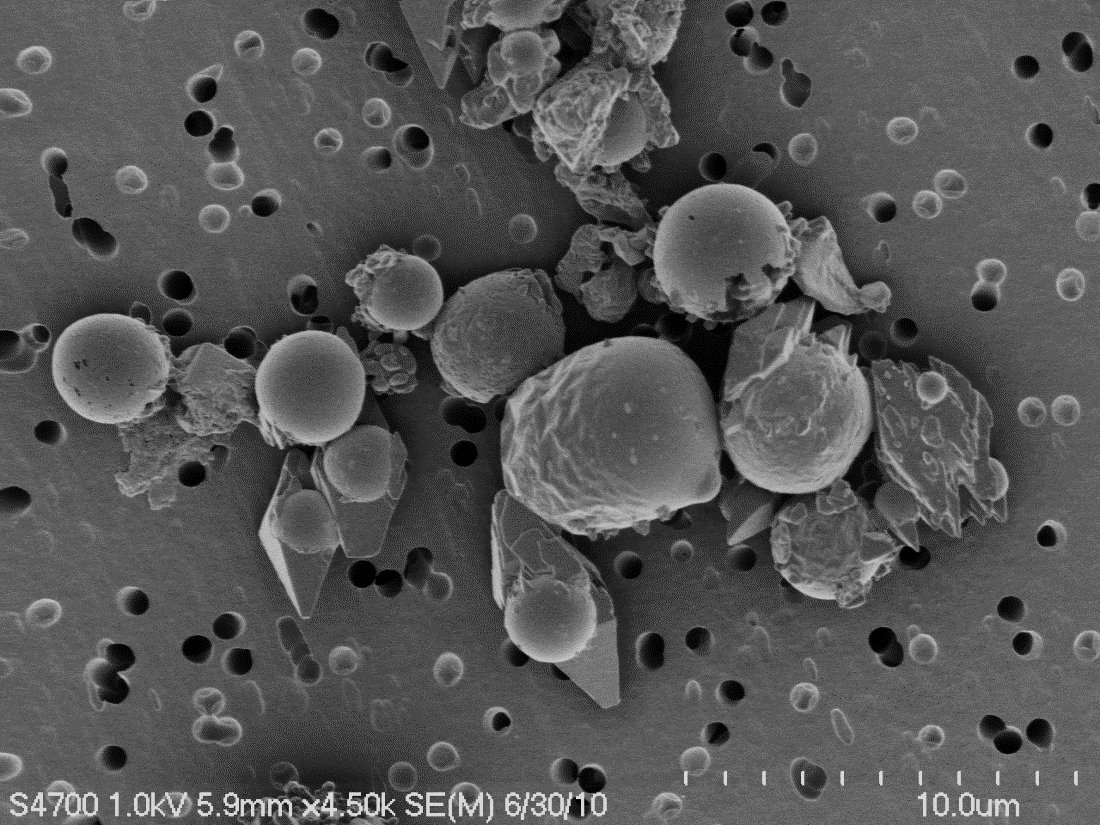


Figure S2. Scanning electron micrograph of biogenic S^0^ produced by mutant *Cba. tepidum* strain C3. Both globules and globular sulfur transitioning to bipyramidal sulfur can be observed.

Figure S3. Fluorescence of FM-dyed *Cba. tepidum* S^0^ globules imaged at 2, 6, and 17 hrs post-incubation. In *Cba. tepidum* cultures, globules tend to form all at once and then grow throughout the production stage (rather than new globules being produced over time). Thus, the positive correlation between globule size and fluorescence shows that with increasing time, there is greater fluorescence. In addition, within any given time point (e.g. 2 hr) there is also a positive correlation between globule size and fluorescence. Larger globules, with greater surface area, are capable of adsorbing greater amounts of organics to the surface until it becomes saturated. When fluorescence is normalized to size, there is still an increase over time, suggesting that a combination of globule size (i.e. available surface area for adsorption) and time (i.e. duration exposed to organics) both play a role in the accumulation of the organic surface layer.
